# Prenatal Evaluation of Scrotal Masses: A Systematic Literature Review

**DOI:** 10.1002/pd.6898

**Published:** 2025-09-26

**Authors:** Federica Romanzi, Chiara Di Ilio, Chiara Airoldi, Gloria Anderson, Eleonora Torcia, Francesca Felici, Maria Vittoria Alesi, Rossana Cottone, Giulia Di Marco, Elvira Passananti, Alessandra Familiari, Tullio Ghi, Elisa Bevilacqua

**Affiliations:** ^1^ Department of Women's and Child Health Sciences and Public Health IRCCS A. Gemelli University Polyclinic Foundation Rome Italy; ^2^ Department of Translation Medicine University of Piemonte Orientale Novara Italy; ^3^ Clinic of Obstetrics and Gynecology Catholic University Sacro Cuore Rome Italy

**Keywords:** differential diagnosis, inguinoscrotal hernia, meconium periorchitis, prenatal diagnosis, scrotal masses, testicular torsion, testicular tumor, ultrasound

## Abstract

Evaluation of fetal genitalia is often neglected after determining fetal sex, yet the identification of a scrotal mass may suggest significant underlying conditions requiring specific management. We conducted a systematic literature review, following PRISMA guidelines and registered with PROSPERO (CRD42024559035), on the five most common causes of prenatal scrotal masses: inguinoscrotal hernia (IH), meconium periorchitis (MPO), testicular solid tumors (TST), hydrocele (H), and testicular torsion (TT). A total of 83 cases were included (IH = 31; MPO = 23; TST = 6; H = 10; TT = 13), all diagnosed in the third trimester. IH typically presented as unilateral right‐sided masses (63%), while MPO and H were predominantly bilateral (72% and 100%). TST and TT were mainly unilateral with no side preference. IH and MPO showed larger average diameters (> 35 mm). Additional findings were often associated with IH and MPO. MPO had the highest rates of preterm delivery (48%), neonatal medical support (40%), and urgent surgery (60.86%). IH and TST were usually associated with term deliveries and scheduled surgery (92.31% and 100%). TT showed a high incidence of urgent surgery (61.54%). This review outlines key sonographic features of prenatal scrotal masses to guide differential diagnosis and optimize perinatal care strategies.

## Highlights

- What's already known about this topic?
  ◦ The differential diagnosis of scrotal masses identified during the prenatal period is challenging, although management and outcomes may differ depending on the nature of the mass.
- What does this study add?
  ◦ A detailed sonographic assessment of scrotal masses detected antenatally helps identify cases that require prompt referral to a fetal medicine center optimizing their clinical management.

## 1 Introduction

Prenatal evaluation of fetal genitalia is often limited and typically not performed beyond sex determination during the mid‐trimester anatomy scan. In male fetuses, the scrotal sac does not yet exhibit its definitive appearance at this gestational age [1, 2]. Testicular descent generally begins around 24 weeks of gestation but may occur later, occasionally as late as the eighth month, making conclusive assessment during the anomaly scan difficult [3, 4]. Consequently, the prevalence and characteristics of prenatally detected scrotal masses are poorly documented. Moreover, current clinical guidelines do not provide standardized protocols for ultrasound evaluation of fetal genitalia or for the management of genital anomalies detected during pregnancy.

The scrotum and its internal structures can present a wide range of anomalies, including morphological abnormalities, scrotal masses, testicular pathologies, and epididymal disorders, each with distinct prevalence, morbidity, and potential clinical implications [5, 6, 7, 8, 9, 10, 11].

Studies have shown that the scrotum can be effectively evaluated by ultrasonography in nearly 100% of cases up to 36 weeks of gestation, with reduced accessibility only after 40 weeks [12]. Therefore, a more detailed prenatal definition of scrotal features is both feasible and desirable.

Scrotal masses pose a particular challenge in prenatal differential diagnosis, as their sonographic appearance may be similar across conditions and can vary between examinations. Nonetheless, an accurate prenatal diagnosis is essential for appropriate follow‐up, optimal timing and setting of delivery, and postnatal management [13].

Current literature on prenatal scrotal masses is limited, consisting mainly of case reports and a small number of reviews [2]. The ultrasound characteristics of different scrotal masses remain insufficiently defined, and there is no consensus on management strategies.

In this study, we present a systematic review of the literature focusing on the main conditions responsible for prenatal scrotal masses: inguinoscrotal hernia (IH), meconium periorchitis (MPO), testicular solid tumors (TST), hydrocele (H), and testicular torsion (TT). Our aim is to synthesize the available data to better define the prenatal ultrasonographic features of these conditions and to propose a diagnostic flowchart that may assist clinicians in achieving an accurate diagnosis and, consequently, in guiding appropriate counseling and perinatal management.

To the best of our knowledge, this is the first systematic literature review dedicated to prenatal scrotal masses.

## 2 Methods

### 2.1 Information Sources and Search Strategy

We conducted a systematic search on SCOPUS, PubMed, and Web of Science up to July 2024. Additional studies, registered as “other sources”, were identified by scanning references.

We used MeSH (Medical Subject Heading) terms with the following keyword and word variants, either alone or in combination: “fetal”, “fetus”, “newborn”, “prenatal diagnosis”, “ultrasound diagnosis”, “intrauterine diagnosis”, “scrotal masses”, “inguinoscrotal hernia”, “meconium periorchitis”, “testicular torsion”, “hydrocele”, “testicular tumor” (Table S1).

The search and selection criteria were restricted to articles in English. No time restrictions were applied, and only full‐text articles were considered eligible for inclusion.

The systematic literature review was conducted in accordance with the Preferred Reporting Items for Systematic Reviews and Meta‐Analyses (PRISMA) [14] statement and registered with PROSPERO (CRD42024559035).

Study authors agreed to the protocol before the research was conducted. No further amendments were applied to the information provided at the registration of the protocol.

Study selection process, eligibility criteria, data collection process and risk of bias assessment.

All case reports, reviews, and case series containing the prenatal ultrasonographic description of a scrotal mass were selected. A further evaluation of the selected articles was performed, and those containing relevant data regarding clinical elements and outcomes, as determined by consensus of the three main researchers (FR, CdI and EB), were included in the study. This selection process was carried out to minimize the risk of bias and the variation in the level of information from each selected case.

Quality assessment of the included studies was performed using the National Institutes of Health (NIH) tool for quality assessment of case series, as reported in Table S2. The quality assessment was performed by two researchers (FR, DdI), with disagreements resolved by consulting the senior author (EB). Data were extrapolated independently by two researchers (FR, CdI) into a datasheet and reviewed by a third (EB); discrepancies were resolved by consensus.

When more than one study was published on the same clinical case with identical endpoints, the report containing the most detailed information was included to avoid overlapping populations.

### 2.2 Data Items, Effect Measures and Synthesis Method

To simplify consultation, the included studies were grouped based on the final post‐birth diagnosis (IH, MPO, TST, H, or TT).

The collected information regarding pregnancy and ultrasound characteristics of the scrotal masses included: maternal age, gestational age (GA) at diagnosis, GA at birth, side, size, ascites, presence of testicular/abdominal calcifications, blood flow signal, bowel peristalsis, bowel dilatation, presence of additional findings, additional description of the scrotal mass, Magnetic Resonance Imaging (MRI), birthweight, Apgar score, and outcome.

Primary outcomes related to sonographic features of the scrotal masses (size, side, homogeneous/heterogeneous/mixed/complex appearance, presence of a cystic/anechoic component, blood flow signal, presence of calcifications/hyperechogenicity, presence of additional findings outside the scrotum, isolated scrotal mass, peristalsis, and the presence of amniotic fluid anomalies reported in the articles as “polyhydramnios” or “mild polyhydramnios”). Structural or anatomical anomalies, as well as documented growth restriction with or without abnormal fetal Doppler findings, were considered among the “additional findings”; anomalies of the amniotic fluid were considered as an independent factor.

Secondary outcomes related to anamnestic element, management, postnatal treatment and outcomes (maternal age, GA at diagnosis, GA at birth, delivery at term, preterm delivery, genetic anomalies, medical support at birth, conservative treatment of the scrotal mass, scheduled surgery, urgent surgical intervention, and neonatal demise).

Cases requiring medical support at birth were considered when it was specifically described, when the Apgar score was lower than 7 at 1 min [15], when the birthweight was < 2000g [16], or when the newborn died within 12 h of birth. The surgical approach was classified as “scheduled” or “urgent” based on the explicit description provided by the authors or as deduced from the detailed reading of the case reports.

### 2.3 Statistical Analysis

Descriptive statistics of the included subjects are reported. Categorical variables are summarized using absolute and relative frequencies, while for numerical variables, the mean and standard deviation (SD) or median and interquartile range [IQR]—composed of first and third quartiles, Q1 and Q3, respectively—are reported, as appropriate. The variables recorded were compared between diagnosis groups using the chi square/Fisher test for categorical variables and Anova models or Kruskal–Wallis test for numerical variables based on normality assumption (checked by Shapiro–Wilks test). Since each study contributed only one or a few subjects to the overall analysis, estimates were performed without pooling the results. Finally, for each variable, analysis was performed excluding missing data, and the number of available data points for each variable was reported.

The significant threshold was set at 0.05 (two tailed), and all analyses were performed using SAS 9.4.

## 3 Results

### 3.1 Review of the Literature—General Characteristics

**FIGURE 1:**
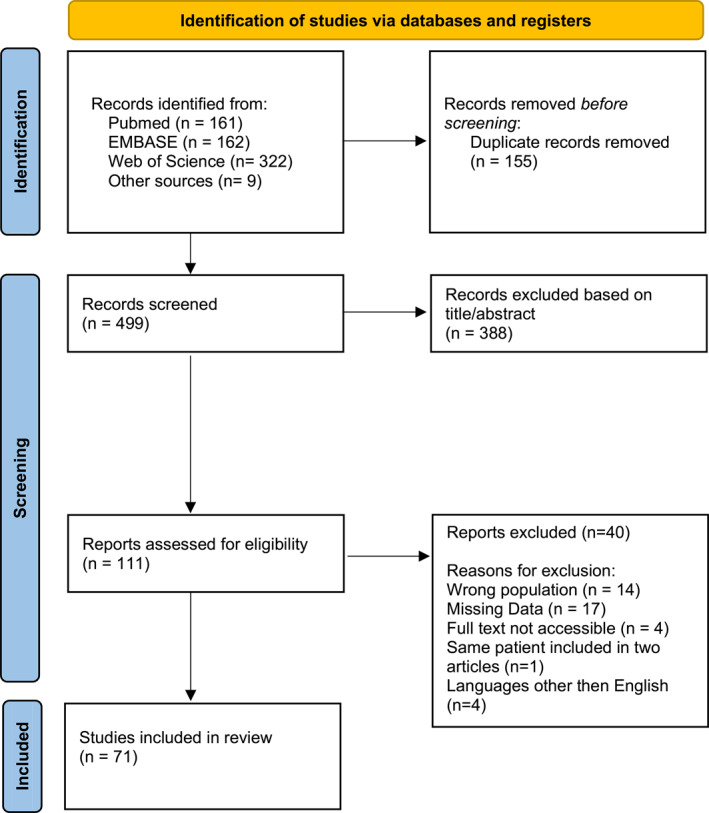
PRISMA 2020 flow diagram for new systematic reviews which included searches of databases and registers only. *Consider, if feasible to do so, reporting the number of records identified from each database or register searched (rather than the total number across all databases/registers). **If automation tools were used, indicate how many records were excluded by a human and how many were excluded by automation tools. Source: Page MJ, et al. BMJ 2021;372:n71. doi: 10.1136/bmj.n71. This work is licensed under CC BY 4.0. To view a copy of this license, visit https://creativecommons.org/licenses/by/4.0/

The literature search of the SCOPUS, PubMed, and Web of Science databases initially retrieved 654 potentially relevant studies. We identified 155 duplicates and excluded 388 results due to content that did not correspond to our purpose, based on title/abstract. We then excluded 40 potentially eligible articles due to incorrect population (e.g., scrotal mass first detected in newborns), missing data, primarily regarding the intrauterine description of the mass or fetal outcome, inaccessibility of the full text, the same patient being included in two articles, and languages other than English. After the full‐text review, 71 studies were included (Figure 1). A table of key studies excluded, along with the reasons, is reported in Table S3.

### 3.2 Review of the Literature—Synthesis of the Results

The analysis included 83 cases from 71 articles, distributed among the following categories: 31 IH (37.3%) [17, 18, 19, 20, 21, 22, 23, 24, 25, 26, 27, 28, 29, 30, 31, 32, 33, 34, 35, 36, 37, 38, 39, 40, 41, 42, 43, 44, 45], 23 MPO (27.8%) [46, 47, 48, 49, 50, 51, 52, 53, 54, 55, 56, 57, 58, 59, 60, 61, 62, 63, 64], 6 TST (7.2%) [65, 66, 67, 68, 69, 70], 10 H (12%) [71, 72, 73, 74, 75, 76, 77], and 13 TT (15.7%) [78, 79, 80, 81, 82, 83, 84, 85, 86, 87]. The descriptions of the included articles are available in Tables [Link], [Link], [Link], [Link], [Link].

The mean maternal age was 29.18 ± 6.02, with no statistically significant differences among the groups (*p* = 0.2444).

Diagnosis occurred earliest for MPO and latest for H (mean GA 32.55 ± 3.28 weeks and 37.50 ± 3.10 weeks, respectively; *p* = 0.0037).

The side of the lesion was described in 80 out of the 83 cases. Bilateral involvement was most common in H (100%) and in MPO (72.73%), whereas unilateral involvement predominated in IH (89.66%) and TT (76.92%) (*p* < 0.0001). IH showed a side predominance having a presentation on the right in 63.33% of cases, whereas TT showed an equal distribution between right and left sided (38.46% each). TST were always unilateral, with a left‐side predominance (66.67% vs. 33.33%).

The smallest minimum lesion size was observed in TT (17.67 ± 4.04 mm), while the largest maximum size was noted in MPO (51.00 ± 13.37 mm), with both differences being statistically significant (*p* = 0.0260 for minimum size, *p* = 0.0016 for maximum size).

**FIGURE 2:**
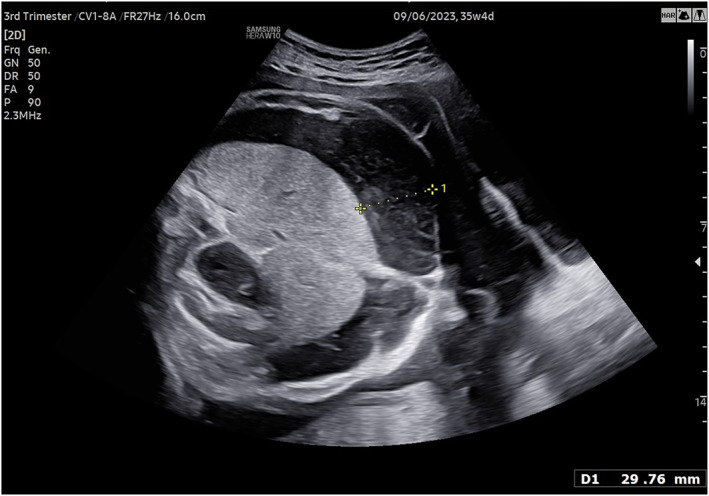
Ultrasound image (Transabdominal scan) of the abdominal ascites in a fetus with MPO.

The presence or absence of ascites was reported in 71 out of 83 cases, occurring in 21.3% of cases overall. It was most frequent in MPO (63%) (Figure 2) and absent in H and TT (*p* < 0.0001).

The presence or absence of testicular or abdominal calcifications/hyperechogenicity was described in 75 cases and was more common in MPO (90.48%) and TT (75%), with a significant difference among groups (*p* < 0.0001).

Blood flow signal data, assessed using Color Doppler examination, were available for 51 cases. Among these, the signal was observed in all cases of TST (100%) and in a portion of IH cases (36.36%). Conversely, no blood flow signal was detected in cases of MPO, H, or TT (*p* = 0.0005).

Bowel peristalsis was described in 75 out of 83 cases and was exclusively observed in IH (86.21%), suggesting its specificity as a diagnostic marker for this condition (*p* < 0.0001).

Data on bowel dilatation were available in 68 cases. A certain degree of dilatation occurred in IH (18.18%), MPO (27.78%), and TST (20.00%), but no statistically significant difference was noted among the groups (*p* = 0.1302).

Scrotal masses were isolated in 50 out of 83 fetuses (60.24%). H showed a low rate of additional findings, with 90% of cases being isolated, while all TT cases were isolated (100%). In MPO, additional findings were present in 69.57% of cases, making isolated masses less common (30.43%). TST were often identified as isolated masses (66.67%) rather than associated with additional findings (33.33%). For IH, isolated and non‐isolated masses occurred at similar frequencies (54.84% and 45.16%, respectively). These differences were found to be statistically significant (*p* = < 0.0001).

A cystic/anechoic component or its absence was described for 54 out of 83 cases, being present in all TST (100%), most MPO (94.74%) and TT (92.31%), and the majority of IH (61.11%) (*p* = 0.0123).

Other sonographic features, such as homogeneous appearance and increased amniotic fluid index, were also evaluated, but no statistically significant differences were observed among the groups (*p* = 0.2706 and *p* = 0.0693, respectively).

Table 1 summarizes information about sonographic features.

**TABLE 1:** Sonographic features of the main conditions responsible for prenatal scrotal masses.

	*All N = 83*	Inguinoscrotal hernia (IH) *N* = 31	Meconium periorchitis (MPO) *N* = 23	Testicular solid tumors (TST) *N* = 6	Hydrocele (H) *N* = 10	Testicular torsion (TT) *N* = 13	*p*‐value
Side	*N* = 80	*N* = 30	*N* = 22	*N* = 6	*N* = 9	*N* = 13	
Bilateral *N* (%)	32 (40.00)	4 (13.33)	16 (72.73)		9 (100.00)	3 (23.058)	< 0.0001
Left *N* (%)	18 (22.50)	7 (23.33)	2 (9.09)	4 (66.67)		5 (38.46)	
Right *N* (%)	30 (37.50)	19 (63.33)	4 (18.18)	2 (33.33)		5 (38.46)	
Min size (mm)	*N* = 35	*N* = 23	*N* = 8	*N* = 3	*N* = 0	*N* = 1	
Mean ± SD	34.40 ± 14.52	37.17 ± 13.29	36.13 ± 14.42	17.67 ± 4.04		7.00	0.0260
Max size (mm)	*N* = 41	*N* = 26	*N* = 9	*N* = 5	*N* = 0	*N* = 1	
Mean ± SD	45.12 ± 18.40	48.77 ± 17.35	51.00 ± 13.37	22.80 ± 5.63		9.00	0.0016
Ascites	*N* = 71	*N* = 27	*N* = 20	*N* = 6	*N* = 6	*N* = 12	
Yes *N* (%)	15 (21.13)	1 (3.70)	13 (65.00)	1 (16.67)	0 (0.00)	0 (0.00)	< 0.0001
Testicular/Abdominal calcifications/hyperechogenicity	*N* = 75	*N* = 31	*N* = 21	*N* = 5	*N* = 6	*N* = 12	
Yes *N* (%)	37 (49.33)	7 (22.58)	19 (90.48)	2 (40.00)	0 (0.00)	9 (75.00)	< 0.0001
Blood flow signal	*N* = 51	*N* = 22	*N* = 9	*N* = 3	*N* = 5	*N* = 12	
Yes *N* (%)	11 (21.57)	8 (36.36)	0 (0.00)	3 (100.00)	0 (0.00)	0 (0.00)	0.0005
Hydrocele	*N* = 78	*N* = 30	*N* = 19	*N* = 6	*N* = 10	*N* = 13	
Yes *N* (%)	44 (56.41)	4 (13.33)	18 (94.74)	2 (33.33)	10 (100.00)	10 (76.92)	< 0.0001
Bowel peristalsis	*N* = 81	*N* = 29	*N* = 23	*N* = 6	*N* = 10	*N* = 13	
Yes *N* (%)	25 (30.86)	25 (86.21)	0 (0.00)	0 (0.00)	0 (0.00)	0 (0.00)	< 0.0001
Bowel dilatation	*N* = 68	*N* = 22	*N* = 18	*N* = 5	*N* = 10	*N* = 13	
Yes *N* (%)	10 (14.71)	4 (18.18)	5 (27.78)	1 (20.00)	0 (0.00)	0 (0.00)	0.1302
Additional findings	*N* = 83	*N* = 31	*N* = 23	*N* = 6	*N* = 10	*N* = 13	
Yes *N* (%)	33 (39.76)	14 (45.16)	16 (69.57)	2 (33.33)	1 (10.00)	0 (0.00)	< 0.0001
Isolated mass	*N* = 83	*N* = 31	*N* = 23	*N* = 6	*N* = 10	*N* = 13	
Yes *N* (%)	50 (60.24)	17 (54.84)	7 (30.43)	4 (66.67)	9 (90.00)	13 (100.00)	< 0.0001
Homogeneous appearance	*N* = 31	*N* = 18	*N* = 6	*N* = 1	*N* = 0	*N* = 7	
Yes *N* (%)	4 (12.90)	1 (5.56)	2 (33.33)	0		1 (16.67)	0.2706
Cystic anechoic component	*N* = 33	*N* = 18	*N* = 4	*N* = 4	*N* = 0	*N* = 7	
Yes *N* (%)	21 (63.64)	11 (61.11)	0	4 (100.00)		6 (85.71)	0.0123
Increased amniotic fluid	*N* = 83	*N* = 31	*N* = 23	*N* = 6	*N* = 10	*N* = 13	
Yes *N* (%)	16 (19.28)	7 (22.58)	8 (34.78)	0 (100.00)	1 (10.00)	0 (0.00)	0.0693
MRI	*N* = 75	*N* = 30	*N* = 16	*N* = 6	*N* = 10	*N* = 13	
Yes *N* (%)	6 (8.00)	3 (10.00)	2 (12.50)	1 (16.67)	0 (0.00)	0 (0.00)	0.5214

Abbreviations: MRI = Magnetic Resonance Imaging, N = Number, SD = Standard Deviation.

Genetic investigations were performed in 54 cases, revealing a 11.11% rate of genetic anomalies, limited to the IH (20%) and MPO (7.14%) groups. However, this difference did not reach statistical significance (*p* = 0.4596).

Regarding perinatal outcomes, birthweight was significantly lower in MPO (2759.32 ± 634.28 g) compared with H and TT (*p* = 0.0031). The lowest rate of term delivery was observed in cases with MPO (52.17%). Notably, no cases of preterm birth were reported among the groups of TST, H, and TT.

Data on Apgar scores, available for 39 cases, showed no significant differences among groups (*p* = 0.0896).

Type of treatment was available for 79 newborns. Non‐surgical management was reported in 23.08% of cases overall, with the highest rates for H (100%) and for TT (23.08%). Scheduled surgery was most common in TST (100%) and IH (92.31%), while urgent intervention was frequent in TT (61.54%) and MPO (60.86%) (*p* < 0.0001).

Table 2 summarizes information regarding anamnestic elements, management, and outcomes.

**TABLE 2:** Anamnestic characteristics, management and outcomes of prenatally diagnosed scrotal masses.

	All *N* = 83	Inguinoscrotal hernia (IH) *N* = 31	Meconium periorchitis (MPO) *N* = 23	Testicular solid tumors (TST) *N* = 6	Hydrocele (H) *N* = 10	Testicular torsion (TT) *N* = 13	*p*‐value
Maternal age (years)	*N* = 71	*N* = 29	*N* = 19	*N* = 3	*N* = 10	*N* = 10	
Mean ± SD	29.18 ± 6.02	30.17 ± 5.77	27.47 ± 7.13	35.00 ± 3.61	28.10 ± 5.49	28.90 ± 4.63	0.2444
GA at diagnosis (weeks)	*N* = 82	*N* = 31	*N* = 23	*N* = 6	*N* = 10	*N* = 12	
Mean ± SD	34.46 ± 4.08	34.49 ± 4.80	32.55 ± 3.28	34.33 ± 4.23	37.50 ± 3.10	35.56 ± 2.20	0.0037
Birth weight (grams)	*N* = 63	*N* = 26	*N* = 19	*N* = 4	*N* = 5	*N* = 9	
Mean ± SD	3107.38 ± 653.30	3071.42 ± 610.65	2759.32 ± 634.28	3150.00 ± 412.55	3612.00 ± 171.23	3648.89 ± 612.57	0.0031
Apgar < 7	*N* = 39	*N* = 18	*N* = 12	*N* = 1	*N* = 1	*N* = 7	
Yes *N* (%)	9 (23.08)	3 (16.67)	6 (50.00)	0 (0.00)	0 (0.00)	0 (0.00)	0.0896
Term delivery	*N* = 76	*N* = 30	*N* = 23	*N* = 6	*N* = 8	*N* = 9	
Yes *N* (%)	61 (80.26)	26 (86.67)	12 (52.17)	6 (100.00)	8 (100.00)	9 (100.00)	0.0029
Genetic anomalies	*N* = 54	*N* = 25	*N* = 14	*N* = 5	*N* = 9	*N* = 1	
Yes *N* (%)	6 (11.11)	5 (20.00)	1 (7.14)^a^	0 (0.00)	0 (0.00)	0 (0.00)	0.4596
Medical support at birth	*N* = 80	*N* = 29	*N* = 22	*N* = 6	*N* = 10	*N* = 13	
Yes *N* (%)	16 (20.00)	6 (20.69)	9 (40.91)	0 (0.00)	0 (0.00)	1 (7.69)	0.0339
Type of treatment	*N* = 79	*N* = 26	*N* = 23	*N* = 6	*N* = 10	*N* = 13	
Conservative *N* (%)	18 (23.08)	1 (3.85)	4 (17.39)	0	10 (100.00)	3 (23.08)	< 0.0001
Scheduled surgery *N* (%)	37 (46.83)	24 (92.31)	5 (21.73)	6 (100.00)	0	2 (15.38)	
Urgent surgical intervention *N* (%)	23 (29.11)	1 (3.85)	14 (60.86)	0	0	8 (61.54)	

Abbreviations: GA = Gestational age, N = Number, SD = Standard Deviation.

^a^ Only fetuses with a homozygous mutation for Cystic Fibrosis were considered.

## 4 Discussion

### 4.1 Main Findings and Comparison With Existing Literature

**FIGURE 3:**
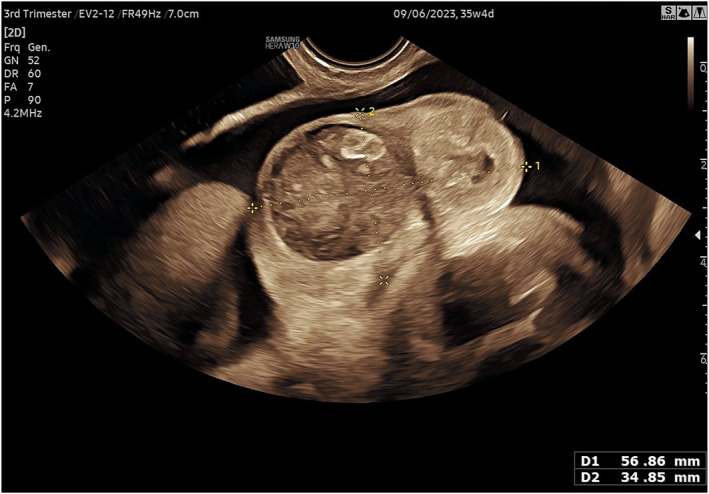
Ultrasound image (Transabdominal scan) of the scrotum in a fetus with MPO.

According to our review, scrotal masses are almost exclusively diagnosed in the third trimester, with the earliest diagnoses observed in the MPO group, likely due to associated findings that draw attention during routine ultrasound. A unilateral presentation was strongly indicative of IH, TT, or TST, with IH being predominantly right‐sided, consistent with literature attributing this to delayed descent and obliteration of the right processus vaginalis, making right‐sided hernias twice as common in the pediatric population [88]. Conversely, MPO and H typically presented bilaterally; however, their diagnosis cannot be excluded a priori in cases of unilateral presentation. Our findings show that larger scrotal masses (> 35 mm), whether unilateral or bilateral, are suggestive of IH or MPO (Figure 3). Morphometric data on fetal male genitalia are sparse [3, 84, 89, 90], but Pinette et al. reported a mean transverse scrotal diameter between 20 and 40 mm from weeks 30 to 41 [90]. We infer that measurements exceeding half this range per hemiscrotum may be abnormal. TST typically exhibits near‐normal measurements, while data on TT and H remain limited.

When additional anomalies are identified, MPO or IH is the most likely diagnoses. MPO was associated with anomalies in 69.57% of cases—most frequently ascites, but also bowel dilatation and abdominal calcifications [46, 48, 50, 51, 57, 61, 63]. These findings may reflect the pathogenesis of meconium ascites. In contrast, associated anomalies in IH cases were often unrelated to the hernia itself, instead reflecting broader chromosomal or syndromic conditions [17, 18, 25, 26, 28, 31, 34, 35, 37, 38, 41].

Genetic abnormalities were most common in the IH group (20%), often with poor outcomes such as perinatal demise [26, 31, 34, 37, 41]. Notably, all IH cases with genetic diagnoses had additional sonographic anomalies, suggesting that isolated IH is generally reassuring. In the MPO group, genetic anomalies were less frequent (7.14%), with only one confirmed case of cystic fibrosis (CF) [57]. While there is a reported association between meconium peritonitis and CF, in‐utero diagnoses of MPO are linked to CF in only 8.3% of cases, compared to 15%–25% when meconium peritonitis occurs after birth [90]. When MPO is diagnosed postnatally, bowel perforation is more commonly related to inspissation of the meconium, whereas vascular causes leading to ischemia or mechanical obstructions, such as volvulus, are more frequent when detected prenatally [90, 91].

Polyhydramnios was common in both MPO (34.78%) and IH (22.58%) cases, though amniotic fluid index values were seldom reported.

Regarding prenatal sonographic features, MPO masses were often hyperechoic, described as testicular calcifications [46, 47, 48, 49, 50, 51, 52, 53, 54, 55, 56, 57, 59, 64], while TT could present with a rounded echogenic mass suggestive of testicular ischemia. Color Doppler studies showed absent flow in MPO, present flow in TST, and low or absent flow in IH, TT, and H. A diagnostic triad for MPO (calcifications, absent blood flow, and hydrocele) has been reported, but it was only present in 39.13% of cases [57].

Fluid characteristics varied; cystic masses were linked to TST or IH, while hydrocele‐like free‐fluid was typical of MPO. A concentric fluid layering pattern was specific to TT (“double ring hemorrhage” image) [91]. Bowel peristalsis within the scrotum, highly indicative of IH, was visible in 86.21% of IH cases, often on repeat examination, highlighting the utility of serial ultrasound in ambiguous cases [20, 22, 29, 35, 36, 40].

Despite diagnostic uncertainty, MRI is underused (only 8%), and its clinical utility remains inconclusive [22, 37, 47, 55, 92].

**FIGURE 4:**
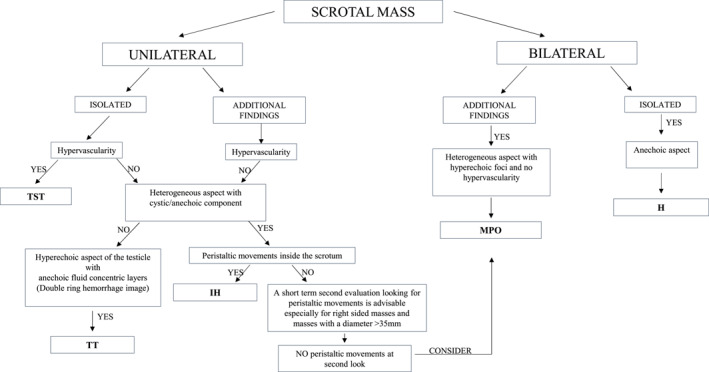
Flowchart for the prenatal differential diagnosis of scrotal masses. H = Hydrocele, IH = Inguinoscrotal hernia, MPO = meconium Periorchitis, TST = Testicular Solid Tumor, TT = Testicular Torsio.

Based on these findings, we propose a diagnostic flowchart to support clinical decision‐making (Figure 4).

Postnatally, MPO cases had the lowest birth weight and highest preterm birth rate (47.83%) [53, 57, 58, 60, 61, 62, 64]. Notably, 4 preterm births were iatrogenic, prompted by deteriorating ultrasound findings [48, 49]. Although low Apgar scores (< 7) were uncommon (9 cases), they were most frequent in the MPO and IH groups, correlating with associated complications. MPO cases also had the highest need for neonatal support (40.91%) [18, 28, 31, 34, 41].

Postnatal management strategies varied by diagnosis. H cases were treated conservatively with favorable outcomes; TST required planned surgery, while 61.54% of TT cases underwent urgent surgery, despite being mostly unilateral [79, 80, 81]. This contrasts with current recommendations suggesting urgent surgery primarily for bilateral TT, reflecting regional differences and clinical uncertainty [93, 94]. IH was managed with scheduled surgery in 92.31% of cases. MPO often necessitates urgent, unscheduled surgery (82.60%), usually due to bowel perforation [46, 48, 49, 50, 51, 53, 57, 58, 61, 62, 63, 64].

## 5 What This Study Adds

Current literature on fetal scrotal anomalies is limited and largely composed of case reports and small retrospective series. To our knowledge, this is the first systematic review to aggregate data on the main prenatal causes of scrotal masses, comparing sonographic characteristics, associated anomalies, and postnatal outcomes. We also propose a novel diagnostic algorithm to aid clinicians in differentiating between these conditions.

Our results suggest that the primary diagnostic goal should be to exclude MPO, as this condition is associated with the highest neonatal morbidity and surgical burden. While scrotal surgery should be reserved for specific indications, abdominal surgery is often required, especially when bowel perforation is suspected. MPO has a generally benign course, with spontaneous resolution of calcifications within 1–5 years if managed conservatively [95, 96, 97]. Awareness of the risk of preterm birth due to polyhydramnios or worsening ultrasound findings is crucial in prenatal counseling.

Although older reports estimate a CF prevalence of 7%–40% in MPO/meconium peritonitis cases, our findings (1 positive case among 14 tested, 7%) align with recent data showing a < 10% CF prevalence in prenatally diagnosed MPO [91, 98, 99, 100]. Therefore, prenatally diagnosed MPO should be considered primarily as a result of mechanical obstruction (atresia, volvulus, imperforate anus, etc.), though CF should be excluded when feasible. This distinction is essential during prenatal counseling, potentially influencing decisions regarding pregnancy continuation.

### 5.1 Study Limitations

This review has several limitations. First, the included studies are predominantly retrospective, with a total limited number of cases and inconsistent reporting across studies. There was also heterogeneity in ultrasound terminology and diagnostic criteria, potentially introducing classification bias. Additionally, data on long‐term outcomes remain scarce, and the value of adjunct imaging tools such as MRI has yet to be clearly established.

## 6 Conclusion

Scrotal masses are rare in fetal life and generally have a favorable prognosis, particularly when not associated with other anomalies. It is essential to improve sonographers' skills in evaluating the male genitalia during the third trimester and to enhance their confidence in recognizing and accurately characterizing different types of scrotal masses. Postnatal management can vary significantly depending on the underlying diagnosis.

Increasing awareness of MPO, its management, and its potential outcomes is critical for appropriate prenatal counseling and clinical decision‐making. Early identification, combined with coordinated multidisciplinary care, facilitates timely referral to specialized centers, helping to prevent treatment delays and avoid unnecessary postnatal surgeries.

## Ethics Statement

The authors have nothing to report.

## Consent

The authors have nothing to report.

## Conflicts of Interest

The authors declare no conflicts of interest.

## Supporting information


**Table S1**: Search strategy.

**Table S2**: Quality assessment of included studies.

**Table S3**: Studies excluded and reasons.

**Table S4**: Prenatal inguinoscrotal hernia (IH).

**Table S5**: Prenatal meconium periorchitis (MPO).

**Table S6**: Prenatal scrotal tumors (TST).

**Table S7**: Prenatal hydrocele (H).

**Table S8**: Prenatal testicular torsion (TT).

## Data Availability

The data supporting the findings of this study are available in the supplementary material of this article.
