# Supplementary material for: Prenatal Evaluation of Scrotal Masses: A Systematic Literature Review
Source: Prenat Diagn. 2025 Sep 26;45(13):1711–22. doi: 10.1002/pd.6898 (PMC12692999; doi:10.1002/pd.6898)
Supplement: Supplementary file 1 — Table S1: Search strategy. [file PD-45-1711-s006.docx]

**Table S1**. **Search strategy**

**Facet Analysis**

| Population | Concept | Contest |
| --- | --- | --- |
| fetal  OR  Fetus  OR  fetuses  OR  infant  OR  Infants  OR  newborn  OR  Newborns | “fetal inguinoscrotal hernia”  OR  “incarcerated hernia”  OR “**inguinal hernia**” OR “**Inguinal hernias**” OR “**inguinoscrotal hernia**” OR “**inguinoscrotal hernias**” OR “**scrotal mass**” OR “**scrotal masses**” OR  “**Meconium periorchitis**” OR “**scrotal calcifications**” OR “**testicular mass**” OR “**testicular masses**” OR  “testicular neoplasm”  OR  “testicular neoplasms” OR “testicular tumor”  OR  “testicular tumors” OR “Testis Neoplasm” OR “Testis Neoplasms” OR “testicular cancer”  OR “testicular cancers” OR “testicular hydrocele” OR “Testicular hydroceles” OR “Scrotal Hydrocele” OR “Scrotal hydroceles” OR “testicular torsion” OR “testicular torsions” OR “spermatic cord torsion” | “Intrauterine diagnosis”  OR “Prenatal Diagnoses”  OR “Intrauterine Diagnoses”  OR  “Intrauterine Diagnosis”  OR “Antenatal Diagnosis”  OR “Antenatal Diagnoses”  OR “Prenatal Screening”  OR  “Prenatal screenings”  OR  “Antental screening”  OR  “Antenatal screenings”  OR  “fetal diagnosis”  OR  “fetal diagnoses”  OR  “fetal screening”  OR  “fetal screenings”  OR  “fetal imaging”  OR  “fetal imagings”  OR  “prenatal presentation”  OR  “ultrasound diagnosis”  OR  “ultrasound diagnoses” |

**Pubmed (161**): **((fetal OR Fetus OR fetuses OR infant OR Infants OR newborn OR Newborns) AND ("fetal inguinoscrotal hernia" OR "incarcerated hernia" OR "inguinal hernia" OR "Inguinal hernias" OR "inguinoscrotal hernia" OR "inguinoscrotal hernias" OR "scrotal mass" OR "scrotal masses" OR "Meconium periorchitis" OR "scrotal calcifications" OR "testicular mass" OR "testicular masses" OR "testicular neoplasm" OR "testicular neoplasms" OR "testicular tumor" OR "testicular tumors" OR "Testis Neoplasm" OR "Testis Neoplasms" OR "testicular cancer" OR "testicular cancers" OR "testicular hydrocele" OR "Testicular hydroceles" OR "Scrotal Hydrocele" OR "Scrotal hydroceles" OR "testicular torsion" OR "testicular torsions" OR "spermatic cord torsion")) AND ("Intrauterine diagnosis" OR "Prenatal Diagnoses" OR "Intrauterine Diagnoses" OR "Intrauterine Diagnosis" OR "Antenatal Diagnosis" OR "Antenatal Diagnoses" OR "Prenatal Screening" OR "Prenatal screenings" OR "Antental screening" OR "Antenatal screenings" OR "fetal diagnosis" OR "fetal diagnoses" OR "fetal screening" OR "fetal screenings" OR "fetal imaging" OR "fetal imagings" OR "prenatal presentation" OR "ultrasound diagnosis" OR "ultrasound diagnoses")**

**Scopus (162):** ( TITLE-ABS-KeY ( fetal OR fetus OR fetuses OR infant OR infants OR newborn OR newborns ) AND TITLE-ABS-KEY ( "fetal inguinoscrotal hernia" OR "incarcerated hernia" OR "inguinal hernia" OR "Inguinal hernias" OR "inguinoscrotal hernia" OR "inguinoscrotal hernias" OR "scrotal mass" OR "scrotal masses" OR "Meconium periorchitis" OR "scrotal calcifications" OR "testicular mass" OR "testicular masses" OR "testicular neoplasm" OR "testicular neoplasms" OR "testicular tumor" OR "testicular tumors" OR "Testis Neoplasm" OR "Testis Neoplasms" OR "testicular cancer" OR "testicular cancers" OR "testicular hydrocele" OR "Testicular hydroceles" OR "Scrotal Hydrocele" OR "Scrotal hydroceles" OR "testicular torsion" OR "testicular torsions" OR "spermatic cord torsion" ) AND TITLE-ABS-KEY ( "Intrauterine diagnosis" OR "Prenatal Diagnoses" OR "Intrauterine Diagnoses" OR "Intrauterine Diagnosis" OR "Antenatal Diagnosis" OR "Antenatal Diagnoses" OR "Prenatal Screening" OR "Prenatal screenings" OR "Antental screening" OR "Antenatal screenings" OR "fetal diagnosis" OR "fetal diagnoses" OR "fetal screening" OR "fetal screenings" OR "fetal imaging" OR "fetal imagings" OR "prenatal presentation" OR "ultrasound diagnosis" OR "ultrasound diagnoses" ) )

Web Of Science (322): [**fetal OR Fetus OR fetuses OR infant OR Infants OR newborn OR Newborns (All Fields) and fetal inguinoscrotal hernia OR incarcerated hernia OR inguinal hernia OR Inguinal hernias OR inguinoscrotal hernia OR inguinoscrotal hernias OR scrotal mass OR scrotal masses OR Meconium periarthitis OR scrotal calcifications OR testicular mass OR testicular masses OR testicular neoplasm OR testicular neoplasms OR testicular tumor OR testicular tumors OR testicular hydrocele OR Testicular hydrocele OR Scrotal Hydrocele OR Scrotal hydrocele OR testicular torsion OR testicular torsions (All Fields) and Intrauterine diagnosis OR Prenatal Diagnoses OR Intrauterine Diagnoses OR Intrauterine Diagnosis OR Antenatal Diagnosis OR Antenatal Diagnoses OR Prenatal Screening OR Prenatal screenings OR antenatal screening OR Antenatal screenings OR fetal diagnosis OR fetal diagnoses OR fetal screening OR fetal screenings OR fetal imaging OR fetal imagings OR prenatal presentation OR ultrasound diagnosis (All Fields)**](https://www-webofscience-com.ezproxy.unicatt.it/wos/woscc/summary/2722a5c3-5f69-4734-ab74-0bb900463bd9-0100115939/relevance/1)
