# Supplementary material for: Prenatal Evaluation of Scrotal Masses: A Systematic Literature Review
Source: Prenat Diagn. 2025 Sep 26;45(13):1711–22. doi: 10.1002/pd.6898 (PMC12692999; doi:10.1002/pd.6898)
Supplement: Supplementary file 2 — Table S2: Quality assessment of included studies. [file PD-45-1711-s007.docx]

| Study | 1. Was study question or objective clearly stated? | 2. Was study population clearly and fully described, including case definition? | 3. Were cases consecutive? | 4. Were subjects comparable? | 5. Was intervention clearly described? | 6. Were outcome measures clearly defined, valid, reliable and implemented consistently across all study participants? | 7. Was length of follow-up described? | 8. Were the statistical methods well-described? | 9. Were the results well-described? |
| --- | --- | --- | --- | --- | --- | --- | --- | --- | --- |
| 2024  Liberty | Yes | Yes | Yes | Yes | Yes | YES | Yes | Yes | Yes |
| 2024  Montironi | Yes | Yes | NA | NA | Yes | NA | Yes | NA | Yes |
| 2024  Bin | Yes | Yes | NA | NA | Yes | NA | Yes | Yes | Yes |
| 2024  Seong | Yes | Yes | NA | NA | Yes | Yes | Yes | Yes | Yes |
| 2024  Asfour | Yes | Yes | NA | NA | Yes | NA | No | NA | Yes |
| 2023  Moustafa | Yes | Yes | NA | Yes | Yes | NA | Yes | Yes | Yes |
| 2023  Thuraisamy | Yes | Yes | NA | Yes | Yes | NA | Yes | Yes | Yes |
| 2022  Chen | Yes | Yes | No | Yes | Yes | Yes | Yes | NA | Yes |
| 2022  Cariello | NA | Yes | NA | NA | Yes | NA | No | NA | NA |
| 2021  Yordanov | Yes | Yes | NA | NA | Yes | Yes | Yes | NA | Yes |
| 2021  Kahraman | Yes | Yes | NA | NA | Yes | Yes | No | NA | Yes |
| 2021  Khoury | Yes | Yes | NA | NA | Yes | Yes | No | NA | Yes |
| 2020  Kumar | Yes | Yes | NA | NA | Yes | Yes | Yes | NA | Yes |
| 2019  Luque | Yes | Yes | Yes | Yes | Yes | Yes | Yes | Yes | Yes |
| 2019  Vatta | Yes | Yes | Na | Na | Yes | Yes | Yes | Yes | Yes |
| 2017  Torres-  cepeda | Yes | Yes | NA | NA | Yes | Yes | Yes | Yes | Yes |
| 2016  Kithir | Yes | Yes | NA | NA | Yes | Yes | No | NA | Yes |
| 2015  Ronzoni | Yes | Yes | NA | NA | Yes | Yes | Yes | NA | Yes |
| 2015  Ochiai | Yes | Yes | NA | NA | Yes | Yes | Yes | Yes | Yes |
| 2015  Gonzalez | Yes | Yes | NA | NA | Yes | Yes | Yes | Yes | Yes |
| 2014  Sanhal | Yes | Yes | NA | NA | Yes | Yes | Yes | Yes | Yes |
| 2014  Stupak | Yes | Yes | NA | NA | Yes | Yes | Yes | Yes | Yes |
| 2014  Illescas | Yes | Yes | NA | NA | Yes | Yes | Yes | Yes | Yes |
| 2014  Melcer | Yes | Yes | NA | NA | Yes | Yes | Yes | Yes | Yes |
| 2013  Massaro | Yes | Yes | NA | NA | Yes | Yes | Yes | NA | Yes |
| 2013  Khatib | Yes | Yes | No | Yes | Yes | Yes | Yes | NA | Yes |
| 2012  Bulotta | Yes | Yes | No | Yes | Yes | Yes | Yes | NA | Yes |
| 2011  Maidarti | Yes | Yes | Yes | NA | NA | Yes | Yes | Yes | Yes |
| 2010  Basaran | Yes | Yes | NA | NA | Yes | Yes | Yes | NA | Yes |
| 2010  Thornburg | Yes | Yes | NA | NA | Yes | Yes | Yes | NA | Yes |
| 2010  Ozcimen | Yes | Yes | NA | NA | Yes | Yes | Yes | NA | Yes |
| 2009 Bohlmann | Yes | Yes | NA | NA | Yes | Yes | Yes | NA | Yes |
| 2009  Jeanty | Yes | Yes | No | NA | NA | No | Yes | Yes | No |
| 2009  Cesca | Yes | Yes | NA | NA | NA | NA | No | No | No |
| 2009  Regev | Yes | Yes | Yes | Yes | Yes | Yes | NA | NA | Yes |
| 2008  Frati | Yes | Yes | NA | NA | Yes | Yes | Yes | NA | Yes |
| 2008  Peterson | Yes | Yes | NA | NA | Yes | Yes | Yes | NA | Yes |
| 2007  Caserta | No | Yes | NA | NA | Yes | Yes | Yes | NA | Yes |
| 2007  Wax | Yes | Yes | Yes | NA | NA | Yes | Yes | Yes | Yes |
| 2006  Chen | Yes | Yes | Yes | Yes | Yes | Yes | Yes | Yes | Yes |
| 2005  Ji | Yes | Yes | NA | NA | Yes | Yes | Yes | NA | Yes |
| 2004  Allen | Yes | Yes | NA | NA | Yes | Yes | Yes | NA | Yes |
| 2004  Arena | Yes | Yes | Yes | Yes | Yes | Yes | Yes | Yes | Yes |
| 2004  Sharma | Yes | Yes | NA | NA | Yes | Yes | Yes | NA | Yes |
| 2002  Koh | Yes | Yes | NA | NA | Yes | Yes | Yes | NA | Yes |
| 2002  Gililland | Yes | Yes | Yes | Yes | Yes | Yes | Yes | Yes | Yes |
| 2001  Ricci | Yes | Yes | NA | NA | Yes | Yes | Yes | NA | Yes |
| 2000  Agarwal | Yes | Yes | NA | NA | Yes | Yes | Yes | NA | Yes |
| 2000  Seow | Yes | Yes | NA | NA | Yes | Yes | Yes | NA | Yes |
| 2000  Youssef BA | Yes | Yes | NA | NA | Yes | Yes | Yes | NA | Yes |
| 2000  Olguner | Yes | Yes | NA | NA | Yes | Yes | Yes | NA | Yes |
| 1998  Devesa | Yes | Yes | NA | NA | NA | Yes | Yes | NA | Yes |
| 1997  Kesby | Yes | Yes | NA | NA | Yes | Yes | Yes | NA | Yes |
| 1996  Paladini | Yes | Yes | NA | NA | Yes | Yes | Yes | NA | Yes |
| 1995  Tripp | Yes | Yes | NA | NA | NA | NA | Yes | Yes | Yes |
| 1995  Shipp | Yes | Yes | NA | NA | No | Yes | No | NA | Yes |
| 1995  Hussain | Yes | Yes | NA | NA | Yes | Yes | Yes | NA | Yes |
| 1995  Konje | Yes | Yes | NA | NA | Yes | Yes | Yes | NA | Yes |
| 1993 Sukcharoen | Yes | NA | NA | NA | Yes | NA | NA | NA | Yes |
| 1993  Gross | Yes | NA | NA | NA | Yes | NA | NA | NA | Yes |
| 1992  Meizner | Yes | Yes | NA | NA | Yes | Yes | Yes | NA | Yes |
| 1991  Ober | Yes | Yes | NA | NA | Yes | Yes | Yes | NA | Yes |
| 1989  Petrikovsky | Yes | Yes | NA | NA | NA | No | No | Yes | Yes |
| 1985  Kenney | Yes | Yes | NA | NA | Yes | Yes | Yes | NA | Yes |
| 1984  Hurwitz | Yes | Yes | NA | NA | Yes | Yes | No | No | No |
| 1983  Hubbard | Yes | Yes | NA | NA | Yes | Yes | No | Yes | Yes |
| 1983  Meizner | Yes | Yes | NA | NA | Yes | Yes | No | Yes | Yes |
| 1980  Di Giacinto | Yes | Yes | NA | NA | Yes | Yes | No | Yes | Yes |
| 1979  Miller | Yes | Yes | Yes | Yes | Yes | Yes | No | Yes | Yes |
| 1978  Vanesian | Yes | Yes | Yes | Yes | Yes | Yes | No | No | Yes |
