# Supplementary material for: Prenatal Evaluation of Scrotal Masses: A Systematic Literature Review
Source: Prenat Diagn. 2025 Sep 26;45(13):1711–22. doi: 10.1002/pd.6898 (PMC12692999; doi:10.1002/pd.6898)
Supplement: Supplementary file 3 — Table S3: Studies excluded and reasons. [file PD-45-1711-s005.docx]

**Table S3.** **Studies excluded and reasons**

| **First author** | **Year of Publication** | **Title** | **Reason for exclusion** |
| --- | --- | --- | --- |
| Meena D | 2017 | Inguinoscrotal hernia in infants: Three case reports in ultrasound diagnosis. | Two out of the three infants were female and the male was diagnosed with an inguinoscrotal hernia after delivery |
| Yada K | 2017 | Laparoscopic resection of prenatally detected intra-abdominal testicular teratoma: Report of a neonatal case | Undescended testicle with no anomalies of the scrotal sac |
| Epelboym Y | 2017 | Ultrasound diagnosis of fetal hypospadias: Accuracy and outcomes | No scrotal anomalies reported in the article |
| Veroniki | 2017 | Comparative safety of anti-epileptic drugs during pregnancy: a systematic review and network meta-analysis of congenital malformations and prenatal outcomes | No intrauterine ultrasound description of the scrotal mass  No data regarding pregnancy and outcomes |
| Bargiacchi S | 2017 | Metatropic dysplasia in third trimester of pregnancy and a novel causative variant in the TRPV4 gene | Female fetus |
| Moon NR | 2015 | Prenatal diagnosis of epignathus with multiple malformations in one fetus of a twin pregnancy using three-dimensional ultrasonography and magnetic resonance imaging. | Inguinoscrotal hernia detected after birth |
| Mahendru A | 2014 | Suspected fetal anomalies | Inguinoscrotal hernia diagnosed after birth |
| Khozeimeh N | 2012 | Management of congenital giant inguinal scrotal hernias in the newborn | No intrauterine ultrasounddescription of the scrotal mass  Incomplete data regarding pregnancy and delivery |
| Riaz-Ul-haq M | 2012 | Neonatal testicular torsion; A review article | No intrauterine ultrasounddescription of the scrotal mass |
| Jacobovitis A | 2010 | Intrauterine development, functions and diseases of male reproductive organs | Article in languages other then English – Hungarian |
| Kaye JD | 2008 | Neonatal torsion: a 14-year experience and proposed algorithm for management | No intrauterine ultrasounddescription of the scrotal mass |
| Edwards S | 2008 | Prenatal diagnosis of monosomy 18p involving a jumping translocation | Wrong Population inguinoscrotal hernia developed after birth |
| Balci S | 2008 | A well-documented trisomy 13 case presenting with a number of common and uncommon features of the syndrome | No scrotal anomalies reported |
| Cuervo JL | 2007 | Perinatal testicular torsion: a unique strategy | No intrauterine ultrasound description of the scrotal mass |
| Mesrobian HG. | 2007 | Urologic problems of the neonate: an update | Missing data about intrauterine aspect of scrotal masses, limited data on outcomes and obstetric history |
| Al-Salem AH | 2007 | Intrauterine testicular torsion: a surgical emergency. | No intrauterine ultrasound description of the scrotal mass |
| Redaelli S | 2005 | Severe intrauterine growth restriction and trisomy 15 confined placental mosaicism: a case report and review of literature. | Inguinoscrotal hernia developed one month after birth |
| Arena F | 2005 | A case of bilateral prenatal testicular torsion: Ultrasonographic features, histopathological findings and management | Data included on another article from the same author |
| Mesrobian HG | 2004 | Urologic problems of the neonate | Missing data about intrauterine aspect of scrotal masses, limited data on outcomes and obstetric history |
| Sorensen MD | 2004 | Prenatal bilateral extravaginal testicular torsion-a case presentation. | Wrong population, post natal diagnosis |
| Van der Sluijs JW | 2004 | Prenatal testicular torsion: diagnosis and natural course. An ultrasonographic study. | Missing data about intrauterine aspect of the scrotal mass, limited data on outcomes and obstetric history |
| Calonge WM | 2004 | Neonatal paratesticular neuroblastoma misdiagnosed as in utero torsion of testis | Missing data about intrauterine aspect of the scrotal mass |
| Colombo P. | 1998 | A case of testicular torsion in utero. Discussion of after birth outcomes | Full text not accessible.  Abstract reporting focus on the after birth management |
| Pretorius DH | 1998 | Hydroceles identified prenatally: common physiologic phenomenon? | Missing data about intrauterine aspect of the scrotal mass |
| Giannakopoulos X | 1997 | Six cases of prenatal and neonatal torsion of the spermatic cord | Missing data about intrauterine aspect of the scrotal mass and about outcomes |
| García González JI | 1997 | Hidrocele meconial [Meconial hydrocele] | Article in languages other then English – Spanish |
| Tamada H | 1997 | A case of infant testicular tumor diagnosed ultrasonographically in the prenatal period | Article in languages other then English – Japanese |
| Moslinger D | 1995 | Meconium peritonitis: Intrauterine appearance - postnatal outcom | Article in languages other then English – German |
| Spier L.N | 1995 | Bilateral abdominoscrotal hydrocele: A case report | Incomplete data regarding pregnancy and outcomes. Missing data on the intrauterine aspect of the mass |
| Timón García A | 1995 | Prenatal torsionof the spermatic cord | Full text not accessible |
| Stone KT | 1995 | Management of suspected antenatal torsion: what is the best strategy? | Wrong population, post natal diagnosis |
| Silver RK | 1994 | Congenital malformations subsequent to chorionic villus sampling: outcome analysis of 1048 consecutive procedures. | Missing data about intrauterine aspect of the scrotal mass, limited data on outcomes and obstetric history |
| Pinette | 1994 | Prenatal diagnosis of inguinal hernia by ultrasound | Full text not accessible |
| Cilento BG | 1993 | Cryptorchidism and testicular torsion | Wrong population, post natal diagnosis |
| Brandt MT | 1992 | renatal testicular torsion: principles of management. | No ultrasounddescription |
| Frank R.G | 1991 | Antenatal Sonographic Evidence of a Paratesticular Mass Representing Healed Meconium Peritonitis | Missing data, only post natal description of the mass |
| Flores-Rivera AR | 1991 | Prenatal testicular torsion.Presentation of a case and review of the literature. | Full text not accessible |
| Brown SM | 1990 | Intrauterine spermatic cord torsion in the newborn: sonographic and pathologic correlation | Missing data about intrauterine aspect of the scrotal mass |
| Yankes JR | 1988 | Antenatal diagnosis of meconium peritonitis with inguinal hernias by ultrasonography.Therapeutic implications | Female foetus |
| D'Addario V | 1986 | Management of a fetus affected by a genitourinary tract anomaly (excluding obstructive uropathies) | Missing data about intrauterine aspect of the scrotal mass |
