# Supplementary material for: Prenatal Evaluation of Scrotal Masses: A Systematic Literature Review
Source: Prenat Diagn. 2025 Sep 26;45(13):1711–22. doi: 10.1002/pd.6898 (PMC12692999; doi:10.1002/pd.6898)
Supplement: Supplementary file 4 — Table S4: Prenatal inguinoscrotal hernia (IH). [file PD-45-1711-s001.docx]

|  | ***Maternal age***  ***(years)*** | ***GA***  ***at***  ***diagnosis***  ***weeks***  ***+ days*** | | ***GA***  ***at***  ***birth***  ***weeks***  ***+ days*** | ***Side*** | ***Size***  ***(mm)*** | ***Ascites*** | ***Testicular/***  ***Abdominal***  ***Calcifications/ hyperechogenicity*** | ***Blood***  ***Flow***  ***signal*** | ***Hydrocele*** | ***Bowel peristalsis*** | ***Bowel***  ***Dilatation*** | ***Additional***  ***Findings/***  ***Additional description*** | ***MRI*** | ***Birth weight***  ***(grams)*** | ***Apgar*** | ***Outcome*** |
| --- | --- | --- | --- | --- | --- | --- | --- | --- | --- | --- | --- | --- | --- | --- | --- | --- | --- |
| **2024**  **Montironi** | 39 | 38+6 | 39 | | RT | 40x35 | NA | NO | NO | YES | NO | NO | -Mild BLT pyelectasia  -Mild polyhydramnios  -Mainly solid mass with regular wall, complex  echotexture and a few small echo-free cystic areas | NO | 2890 | 9-10 | Scheduled surgical repair at 15 days because of incarcerated bowel loop |
| **2024**  **Bin** | 40 | 30 | 40 | | BLT | 42x38 | NO | NO | NO | NO | NA | NO | -Growth restriction | NO | 2000 | 7-8-9 | Respiratory distress at birth requiring admission to NICU  Hernia repair after stabilization of the newborn |
| **2024**  **Liberty** | 34 | 29 | 38 | | BLT | 66x47x48 | NO | YES | YES | YES | YES | NO | -Mild polyhydramnios  -hyperechogenic scrotal mass with scattered hypoechoic and hyperechoic  elements surrounded by moderate to large amounts of fluid and testicles displaced laterally. | NO | 3900 | NA | Elective surgical repair 3 weeks post partum. |
| **2024**  **Seong** | 38 | 38 | 39+1 | | LT | 49x28 | NO | NO | NO | NO | YES  During a second evaluation | NO | NO  Complex echogenicity | NO | 3000 | 9-9 | Scheduled surgical repair 4 days after birth.  Continuous projectile vomiting and poor weight gain, led to a diagnosis of hypertrophic pyloric stenosis.  On the 28th day after birth, pyloromyotomy was performed. |
| **2024**  **Asfour** | 44 | 36 | 39 | | RT | 57x34 | NO | NO | NO | NO | YES | NO | NO  heterogeneous consistency and a mild cystic component. | NO | 3140 | 9 – 9 | Uneventful follow up at 6 months.  scheduled hernia repair |
| **2022**  **Chen** | 32 | 39 | 40 | | RT | 46x35x30 | NO | NO | YES | NO | YES  During a second evaluation | YES | Nonhomogeneous in echotexture and  predominantly solid mass with few small echo‑free cystic  Areas.  Surviving co‑twin after selective fetal reduction in dichorionic twins. | YES | 2720 | 10-10 | Scheduled hernia repair at 8 months |
|  | 28 | 36 | 38+3 | | RT | 43x31 | NO | YES | YES | NO | YES | NO | NO | YES | 3200 | 10-10 | Scheduled surgical correction at 1 month |
| **2022**  **Cariello** | NA | 29 | 39 | | LT | 30x35 | NO | YES | NO | YES | NA | NA | NO  Nonhomogeneous echotexture | NO | NA | NA | Surgical hernia repair |
| **2021**  **Yordanov** | 31 | 37 | 39 | | RT | 36x37 | NA | NO | NO | NO | NO | NA | NO  homogeneous ultrasound structure | NO | 3290 | 9-10 | Scheduled surgical correction at 2 months |
| **2021**  **Kahraman** | 30 | 36+5 | 38 | | LT | NA | NO | NO | NO | NO | YES | NO | Pelvic right kidney | NO | 3245 | 9-10 | Scheduled hernia repair |
| **2020**  **Kumar** | 19 | 35+2 | 36+2 | | RT | 38x36x25 | NO | NO | YES | YES | YES | NA | -Growth restriction (<1 percentile)  -Left hydrocele  -Trisomy 18  -mixed echogenic mass with largely solid and a few echo-free areas and regular borders | NO | 1500 | NA | Fetal intrauterine demise |
| **2016**  **Kithir** | 22 | 37 | 39 | | RT | 29x17 | NO | NO | NA | NO | YES | NO | NO  Heterogeneous in  echotexture | NO | 3050 | NA | Spontaneous resolution with no need for surgical intervention.  Bilateral patent processus vaginalis with a small communicating hydrocele |
| **2015**  **Ronzoni** | 34 | 31+5 | 34+5 | | RT | NA | NO | NO | NO | NO | YES | YES | - Abnormal Doppler  - complete fetal AV block  -low anorectal  anomaly with perineal fistula  -mild poli-  hydramnios | NO | 2230 | 3 - 7 | Anoplasty on second day of life, followed by restoration of normal bowel function.  Right inguinal hernia repair anOrchidopexy scheduled after 6 months |
| **2013**  **Massaro** | 30 | 37 | 38 | | RT | 54x46 | NO | YES | NO | NO | YES During a second evaluation | NO | NO  Echogenic mass with mixed echotexture and regular walls  containing few small echo-free cystic areas | NO | 3700 | 8 – 9 | Scheduled surgery on day 10 after birth |
| **2013**  **Khatib** | 29 | 36 | 38+6 | | BLT | 42x33x28 | NO | NO | YES | NO | YES | NO | Mild  Polihydramnios | NO | 3160 | 9-10 | Scheduled surgical correction at 1 month |
|  | 25 | 24 | NA | | NA | NA | NO | YES | NA | NO | YES | NO | NO  Swollen, enlarged  scrotum with an  echogenic mass inside the scrotum | NO | NA | NA | Scheduled surgical correction at 1 month |
| **2010**  **Basaran** | 28 | 28 | 40 | | LT | NA | NO | YES | NA | NO | YES | NA | -Spondylo costal dysostosis  -mild pyelectasia | NO | 2900 | 9 – 8 - 6 | Fetus affected by Jarcho-Levin syndrome  Died at 3 days for respiratory complication |
| **2010**  **Thornburg** | NA | 40+6 | 41 | | RT | 80X53 | NO | NO | NO | NO | YES | NO | Oligohydramnios | NO | NA | NA | Scheduled surgical repair on day 4 |
| **2010**  **Ozcimen** | 30 | 35 | 39 | | RT | 37X27 | NO | NO | NO | NO | YES | NO | NO | NO | 3500 | 9-10 | Scheduled surgical repair at 12 days |
| **2009 Bohlmann** | 38 | 33+5 | 35  Sp. Lab | | RT | NA | NO | NO | NA | NO | YES | NA | -multiple anomalies  - Trisomy 18  - Polihydramnios | NO | NA | NA | Died within few hours |
| **2008**  **Frati** | 29 | 35 | 40+4 | | RT | 55 | NO | YES | YES | NO | YES  During a second evaluation | NA | -Heterogeneous mass containing echogenic areas  - BLT mild hydronephrosis | NO | 3650 | NA | Scheduled surgery on day 4  Uneventful follow up at 3 months |
| **2007**  **Caserta** | 29 | 21 | 37 | | RT | 33x30 | NO | NO | NO | NO | YES  During a second evaluation | NO | NO  Solid-mass with  scattered small echofree/ cystic  components  characterized by a  Complex echogenicity | NO | 3300 | 7 - 10 | Scheduled surgical correction during the neonatal period |
| **2005**  **Ji** | 30 | 36 | 37 | | LT | 42x50x38 | YES | NO | YES | NO | NO | NA | - Polihydramnios  - Solid predominantly  echogenic without a cyst  - deformity of LT foot | YES | 3200 | NA | multiple joint contractures  Failed To Thrive (FTT) and died at 5 days |
| **2004**  **Allen** | 26 | 34 | >37 | | LT | 36x30 | NO | NO | NA | NO | YES | YES | - Echogenic bowel  - complex mass | NO | 2676 | 8 – 9 | Scheduled surgical correction at 3 weeks of large LT IH and a small RT IH  Fetus affected by cystic fibrosis |
| **2004**  **Sharma** | 30 | 34 | 36  Sp. R.O.M. | | RT | 32x27 | NA | NO | NO | NO | NO | NO | NO  Solid  nonhomogeneous  echogenicity | NO | 2460 | NA | Scheduled surgical correction on day 4 |
| **1997**  **Kesby** | 28 | 36 | 39 | | RT | 46x51 x43 | NO | NO | NA | NO | YES  During a second evaluation | YES | NO  Complex echogenicity  of the mass,  predominantly solid scattered with small cystic components | NO | 3580 | 6 – 9 | Surgical correction on 13th day.  Complicated recovery for appearance of controlateral indirect inguinal hernia requiring second surgery on day 28th |
| **1996**  **Paladini** | 26 | 30 | 37 | | RT | 42 | NO | NO | NA | NO | YES | NA | -Multiple anomalies  -omphalocele  -Trisomy18  -Polihydramnios  -Mixed echotexture, with small fluid-filled  cyst-like spaces  within it | NA | 2300 | NA | Died 3 hours after delivery |
| **­­­­­­1995**  **Shipp** | 30 | > 40 | >40 | | RT | 40 | NO | NO | YES | NO | YES | NO | -Torsion of the left testis with hydrocele  -RT complex mass | NO | NA | NA | Surgical correction in the neonatal period |
| **1995**  **Hussain** | 32 | 41+1 | 41+3 | | BLT | 110x90x80 | NA | NO | NO | NO | YES | NA | NO  small cystic tubular spaces inside the mass | NO | 4096 | 8-9 | Scheduled surgical correction at 2 days |
| **1992**  **Meizner** | 21 | 33 | >37 | | LT | 65x56x54 | NO | NO | NA | NO | YES | NO | NO  Echo-free cyst-like structure | NO | 3400 | NA | Reduction after birth and scheduled surgical correction on day 4. |
| **1991**  **Ober** | 23 | 41 | 41 | | RT | 59x48 | NO | NO | NA | NA | YES | NO | NO | NO | 3770 | 8-9 | Manual reduction of the hernia and scheduled surgical repair on day 1 of life |

***Abbreviations****: AV= atrio-ventricular, BLT = Bilateral, GA = gestational age, IH= Inguinoscrotal hernia, LT= Left, MRI= Magnetic Resonance Imaging, NA= Not Available, NICU= neonatal intensive care unit, RT= Right, Sp Lab= Spontaneous labour, Sp. R.O.M.= Spontaneous Rupture Of Membrane*
