# Supplementary material for: Prenatal Evaluation of Scrotal Masses: A Systematic Literature Review
Source: Prenat Diagn. 2025 Sep 26;45(13):1711–22. doi: 10.1002/pd.6898 (PMC12692999; doi:10.1002/pd.6898)
Supplement: Supplementary file 5 — Table S5: Prenatal meconium periorchitis (MPO). [file PD-45-1711-s003.docx]

|  | **Maternal age**  **(years)** | **GA**  **at**  **diagnosis**  **weeks**  **+ days** | **GA**  **at**  **birth**  **weeks + days** | **Side** | **Size**  **(mm)** | **Ascites** | **Testicular/**  **abdominal calcifications /hyperechogenicity** | **Blood**  **Flow**  **signal** | **Anechoic component** | **Bowel**  **peristalsis** | **Bowel**  **Dilatation** | **Additional**  **Findings/**  **Additional description** | **MRI** | **Birth weight**  **(grams)** | **Apgar** | **Outcome** |
| --- | --- | --- | --- | --- | --- | --- | --- | --- | --- | --- | --- | --- | --- | --- | --- | --- |
| **2024**  **Our Case** | 38  26  (ovocyte donor) | 32 | 35+5 | LT | 34X46 | YES | YES | NO | YES | NO | NO | -Mild polyhydramnios  -Heterogeneous appearance of the scrotal mass | NO | 3100 | 6 - 9 | Non invasive respiratory support at birth  Ileum perforation and mild right inguinal hernia  Ileostomy during urgent surgery and scheduled right orchidopexy 15 days later  **CF NEGATIVE** |
| **2023**  **Moustafa** | 35 | 32 | 37 | BLT | 78X66 | NO | YES | NA | NO | NO | NO | -Enlarged scrotum, with a large volume of echogenic fluid,debris,and calcifications  -liver calcifications | NO | 3210 | NA | Exploratory laparotomy was performed on day of life 1 after suspected pneumoperitoneum. Meconium and meconium pseudocyst were noted without  overt bowel perforation. Small bowel resection was performed  with ileostomy secondary to extensive adhesions.  On day of life 42, ileostomy reversal was performed  with resection of 10 cm of terminal Ileum and right colon  after identifying perforation sites in these segments,  followed by side-by-side ileocolic anastomosis.  Maternal prenatal  screening was negative for CF.  **CF NA** |
| **2021**  **Khoury** | NA | 34+5 | 39 | RT | 54X44X26 | NO | YES | NA | YES | NO | NO | NA  Heterogeneous aspect of the scrotal mass | YES | NA | NA | Intraoperative diagnosis of meconium periorchitis, ligation of the ernia sac.  Scheduled surgery  **CF NA** |
| **2019**  **Luque** | 32 | 34 | 39 | RT | NA | NO | NO | NA | YES | NO | NO | NO  Heterogeneous aspect of the scrotal mass | NO | NA | NA | Abdominal distension at day 5 with conservative treatment.  Scheduled surgical removal of the scrotal mass at 25 days  **CF NOT TESTED** |
|  | 20 | 33+4 | 34+3 | BLT | 44x30  33 | YES | YES | NO | YES | NO | NO | NA  Scrotal hydrocele with calcifications | NO | 2330 | NA | Urgent surgery 20 hours after birth, ileal perforation requiring ileostomy + bilateral orchidopexy  **CF NEGATIVE** |
|  | 32 | 35+4 | 35 | BLT | NA | YES | YES | NA | YES | NO | YES | Polyhydramnios  Scrotal hydrocele with calcifications | NA | NA | NA | Urgent surgery due to ileal perforation repaired with anastomosis and contestual bilateral orchidopexy.  Dehiscence of the scrotal wound after 15 days requiring scrotal sac reconstruction.  **CF NEGATIVE** |
| **2017**  **Torres-**  **cepeda** | 20 | 36 | 36 | BLT | NA | YES | YES | NO | YES | NO | NA | -Polyhydramnios  - Hydrocele with calcifications | NO | 3000 | 6 - 8 | Distended abdomen with subsequent paracentesis with drainage of 150mL of meconial material.  Urgent surgical bowel resection and anastomosis, evacuation of the scrotal sac and bilateral inguinal hernia repair.  **CF NA** |
| **2015**  **Ochiai** | 35 | 28 | 37 | BLT | NA | YES | YES | NO | YES | NO | TRANSIENT | NO  hydrocele and peritesticular calcification | NO | 2545 | 8 - 9 | Bowel perforation. Urgent surgical treatment at 3 days. Conservative follow up of the scrotal mass.  **CF NA** |
| **2014**  **Sanhal** | 23 | 33 | 38 | NA | NA | YES | YES | NA | NA | NO | YES | NO | NO | 3325 | 6-8 | Urgent surgery on day 3 because of bowel perforation: bowel resection and anastomosis and scrotal meconium evacuation  **CF Heterozygous** |
| **2014**  **Stupak** | 32 | 29 | 40 | BLT | 61 X 19  32 | NO | YES | NO | YES | NO | NA | NO  enlarged scrotum with echogenic foci | NA | 3850 | 10 - 10- 10 | Clinical follow up  **CF NEGATIVE** |
| **2011**  **Maidarti** | 35 | 29 | 35 | BLT | 30X30 | YES | YES | NA | NA | NO | NO | Doppler  Anomalies  homogeneous appearance of the meconium filling the scrotum with hyperechogenicity | NO | 2050 | 5 - 8 | Pregnancy complicated by severe preeclampsia.  Abdominal distension and respiratory distress at 24 hours led to peritoneal drainage of meconium and urgent primary ileostomy on day 3.  Conservative management of a small scrotal hernia.  **CF NA** |
| **2009**  **Jeanty** | 16 | 36 | >37 | BLT | NA | NO | YES | NO | YES | NO | NO | -IUGR  -Bilateral hydrocele with calcifications | NA | 3080 | NA | Uneventful follow up  **CF NOT TESTED** |
| **2009**  **Cesca** | 32 | 32 | 40 | BLT | 55x45 | NO | YES | MILD | NA | NO | NA | NA  Large fluid-filled scrotum, cystic aspect with septa | YES | 4000 | NA | Scheduled surgical removal of the scrotal mass.  **CF NEGATIVE** |
| **2009**  **Regev** | NA | 39 | >39 | BLT | NA | NA | YES | NA | YES | NO | NO | NA  Bilateral hydrocele with calcifications | NO | NA | NA | Excision of the right paratesticular mass at 5 months  **CF NEGATIVE.** |
|  | NA | 36 | >37 | BLT | 46X39 | NA | YES | NA | YES | NO | NO | NA  Fluid-filled scrotum with calcifications | NA | 2860 | NA | Uneventful follow up  **CF NA** |
| **2007**  **Wax** | 22 | 34 | 35 | BLT | NA | YES | YES | NA | YES | NO | YES | -Echogenic bowel  -Bilateral hydrocele with calcifications | NO | 2330 | NA | Colon perforation  Urgent surgery with colon resection and colostomy.  Conservative management of the scrotal mass.  **CF POSITIVE** |
| **2002**  **Koh** | NA | 28 | 31 | BLT | NA | YES | NA | NA | YES | NO | NO | NA | NO | 1966 | 8 - 8 | Bilateral pneumoscrotum, ileum atresia with proximal perforation.  Urgent surgery with bilateral herniorrhaphy, bowel resection and anastomosis  **CF NA** |
| **2002**  **Gililland** | 25 | 29 | 37 | RT | 45 | NA | YES | NO | YES | NO | NO | -Small right  inguinal hernia  -Initially homogeneous appearance of the meconium filling the hemiscrotum  -Complex echogenicity with calcifications on subsequent evaluation | NA | 3002 | 8 - 9 | Viable, cyanotic, undescended right testicle, meconium filled pouch. Right orchidopexy, excision of mass.  **CF NEGATIVE** |
| **2000**  **Agarwal** | 26 | 31 | 33+3 | RT | NA | YES | NO | NA | YES | NO | NO | Polyhydramnios | NO | 3150 | 6 | Respiratory distress at birth.  Spontaneous resolution  **CF NEGATIVE** |
| **2000**  **Seow** | 37  ICSI* | 30 | 36 | BLT | NA | YES | YES | NA | YES | NO | YES | -Bicorial biamniotic twin pregnancy with meconium peritonitis in one foetus  -heterogeneous hydrocele  -intrabdominal calcifications  -polyhydramnios | NO | 2170 | 8-9 | Assisted ventilation and intubation after birth  Emergency laparotomy was performed on the male twin because of dyspnea. A 0.2-cm perforation was found in the terminal ileum. Ileotomy was performed and closed after 27 days.  Amniocentesis negative |
| **1995**  **Konje** | 23 | 30 | 31 | BLT | NA | YES | NA | NA | YES | NO | YES | --Polyhydramnios  --echoic abdominal cystic mass | NO | 2130 | 2-5 | Urgent CS for altered CTG  Invasive respiratory support at birth  Urgent surgery revealing extended ileal necrosis, short bowel and meconium ileus. Tenual resection and ileostomy were performed.  **CF Heterozygous.**  **Clinical CF POSITIVE** |
| **1993 Sukcharoen** | 22 | 38 | 39 | LT | NA | YES | YES | NA | YES | NO | NA | --Abdominal echogenicity  --polyhydramnios | NO | 2640 | NA | Terminal ileum perforation.  Urgent surgery  **CF NEGATIVE** |
| **1985**  **Kenney** | 17 | 28+5 | 30 | BLT | NA | NO | YES | NA | NA | NO | NA | --Hypospadia  -polyhydramnios | NO | 1670 | NA | Colon perforation, distal microcolon.  Urgent surgery with left inguinal hernia repair and total colectomy.  **CF NEGATIVE** |

**A*bbreviations****: BLT = Bilateral, CF= Cystic Fibrosis, CS= Caesarean section, CTG= cardiotocography, GA = gestational age, ICSI= Intracytoplasmic sperm injection, LT= Left, MRI= Magnetic Resonance Imaging, NA= Not Available, RT= Right*

*ICSI from testicular sperm extraction was required because of a hypospermatogenetic azoospermia due to cancer surgery and radiotherapy in the husband.
