# Supplementary material for: Prenatal Evaluation of Scrotal Masses: A Systematic Literature Review
Source: Prenat Diagn. 2025 Sep 26;45(13):1711–22. doi: 10.1002/pd.6898 (PMC12692999; doi:10.1002/pd.6898)
Supplement: Supplementary file 6 — Table S6: Prenatal scrotal tumors (TST). [file PD-45-1711-s008.docx]

|  | **Maternal**  **age**  **(years)** | **GA**  **at**  **diagnosis**  **weeks**  **+ days** | **GA**  **at**  **birth**  **weeks**  **+ days** | **Side** | **Size**  **(mm)** | **Ascites** | **Testicular/**  **abdominal calcifications/**  **hyperechogenicity** | **Blood**  **Flow**  **signal** | **Hydrocele** | **Bowel**  **peristalsis** | **Bowel**  **Dilatation** | **Additional**  **Findings/**  **Additional description** | **MRI** | **Birth weight**  **(grams)** | **Apgar** | **Outcome** |
| --- | --- | --- | --- | --- | --- | --- | --- | --- | --- | --- | --- | --- | --- | --- | --- | --- |
| **2023**  **Thuraisamy** | 34 | 26 | 37+5 | RT | 25 | YES | YES | NA | YES | NO | YES | Bilateral hydrocele | YES | 2780 | NA | Suspected scrotal tumor based on MRI performed at 32weeks.  Scheduled surgery  Histopathological diagnosis not available |
| **2019**  **Vatta** | NA | 38 | 39 | LT | 20X20 | NO | NO | NA | NA | NO | NA | Multicystic appearance  of the mass | NO | NA | NA | Radical orchiectomy  Histopathological diagnosis: Juvenile granulosa cell tumor |
| **2015**  **Gonzalez** | 39 | 35 | 39+2 | RT | 24 | NO | NO | NA | NO | NO | NO | NO  Multiple anechoic spots inside the scrotum | NO | 2910 | 9-10 | βHCG and alphafetoprotein (AFP) elevated  Surgery 24 hours after birth with right orchiectomy  Histopathological diagnosis: Juvenile granulosa cell tumor. |
| **2014**  **Illescas** | 32 | 36 | 38+2 | LT | 20X25X30 | NO | YES | YES | NO | NO | NO | NO  Smallsonolucent areas inside an heterogeneous scrotal mass | NO | 3710 | NA | Radical orchiectomy two months after birth.  Histopathological diagnosis: Juvenile granulosa cell tumor |
|  |  |  |  |  |  |  |  |  |  |  |  |  |  |  |  |  |
| **2012**  **Bulotta** | NA | 36 | >40 | LT | 15x13 | NO | NO | YES | NO | NO | NO | NO  Multicystic testicular mass | NO | 3200 | NA | βHCG, AFP, Inhibin B and testosterone  Left radical orchiectomy was performed  Histopathological diagnosis: Juvenile granulosa cell tumor |
| **2008**  **Peterson** | NA | 35 | 38+2 | LT | NA | NO | NA | YES | YES | NO | NO | -Dextrocardia -midline liver  -possible asplenia -cystic structure of the scrotal mass  -Bilateral hydrocele | NO | NA | NA | Radical orchiectomy  Histopathological diagnosis: Juvenile granulosa cell tumor |

***Abbreviations:*** *BLT = Bilateral, GA = gestational age, LT= Left, MRI= Magnetic Resonance Imaging, NA= Not Available, RT= Right*
