# Supplementary material for: Prenatal Evaluation of Scrotal Masses: A Systematic Literature Review
Source: Prenat Diagn. 2025 Sep 26;45(13):1711–22. doi: 10.1002/pd.6898 (PMC12692999; doi:10.1002/pd.6898)
Supplement: Supplementary file 7 — Table S7: Prenatal hydrocele (H). [file PD-45-1711-s004.docx]

|  | **Maternal**  **age**  **(years)** | **GA**  **at**  **diagnosis**  **weeks**  **+ days** | **GA**  **at**  **birth**  **weeks + days** | **Side** | **Size**  **(mm)** | **Ascites** | **Testicular/**  **abdominal calcifications** | **Blood**  **Flow**  **signal** | **Hydrocele** | **Bowel**  **peristalsis** | **Bowel**  **Dilatation** | **Additional**  **findings** | **MRI** | **Weight**  **at**  **birth**  **(grams)** | **Apgar** | **Outcome** |
| --- | --- | --- | --- | --- | --- | --- | --- | --- | --- | --- | --- | --- | --- | --- | --- | --- |
| **2006**  **Chen** | 38 | 37 | 38 | BLT | NA | NO | NO | NA | BLT | NO | NO | - Severe bradycardia  - Polyhydramnios | NO | 3590 | 8-9 | Third-degree atrioventricular block and urgent transvenous pacemaker implantation  Positive antinuclear and anti-Ro antibodies in the newborn. |
| **1989**  **Petrikovsky** | 28 | 30 | NA | NA | NA | NA | NO | NO | YES | NO | NO | NO | NO | NA | NA | Spontaneous resolution during intrauterine development |
|  |  |  |  |  |  |  |  |  |  |  |  |  |  |  |  |  |
| **1984**  **Hurwitz** | 32 | 38 | 40 | BLT | NA | NO | NA | NA | BLT | NO | NO | NO | NO | NA | NA | NA |
|  |  |  |  |  |  |  |  |  |  |  |  |  |  |  |  |  |
| **1983**  **Meizner** | 18 | 41 | NA | BLT | NA | NO | NO | NO | BLT | NO | NO | NO | NO | 3600 | NA | No intervention required  Good outcome |
|  |  |  |  |  |  |  |  |  |  |  |  |  |  |  |  |  |
|  | 28 | 37 | NA | BLT | NA | NO | NO | NO | BLT | NO | NO | NO | NO | 3850 | NA | No intervention required  Good outcome |
| **1980**  **Di Giacinto** | 31 | 36 | NA | BLT | NA | NA | NO | NO | BLT | NO | NO | NO | NO | NA | NA | No intervention required  Good outcome |
| **1979**  **Miller** | 23 | >40 | NA | BLT | NA | NA | NA | NA | BLT | NO | NO | NO | NO | NA | NA | No intervention required  Good outcome |
|  | 30 | >40 | NA | BLT | NA | NA | NA | NA | BLT | NO | NO | NO | NO | NA | NA | No intervention required  Good outcome |
| **1978**  **Vanesian** | 29 | 39 | 39 | BLT | NA | NA | NA | NA | BLT | NO | NO | NO | NO | 3370 | NA | No intervention required  Good outcome |
|  | 24 | 37 | 38 | BLT | NA | NO | NO | NA | BLT | NO | NO | undescended testes | NO | 3650 | NA | NA |
|  |  |  |  |  |  |  |  |  |  |  |  |  |  |  |  |  |

***Abbreviations:*** *BLT = Bilateral, GA = gestational age, LT= Left, MRI= Magnetic Resonance Imaging, NA= Not Available, RT= Right*
