# Supplementary material for: Prenatal Evaluation of Scrotal Masses: A Systematic Literature Review
Source: Prenat Diagn. 2025 Sep 26;45(13):1711–22. doi: 10.1002/pd.6898 (PMC12692999; doi:10.1002/pd.6898)
Supplement: Supplementary file 8 — Table S8: Prenatal testicular torsion (TT). [file PD-45-1711-s002.docx]

|  | **Maternal**  **age**  **(years)** | **GA**  **at**  **diagnosis**  **weeks**  **+ days** | **GA**  **at**  **birth**  **weeks + days** | **Side** | **Size**  **(mm)** | **Ascites** | **Testicular/**  **abdominal calcifications/**  **Hyperechogenicity** | **Blood**  **Flow**  **signal** | **Hydrocele** | **Bowel**  **peristalsis** | **Bowel**  **Dilatation** | **Additional**  **findings** | **MRI** | **Birth**  **weight**  **(grams)** | **Apgar** | **Outcome** |
| --- | --- | --- | --- | --- | --- | --- | --- | --- | --- | --- | --- | --- | --- | --- | --- | --- |
| **2014**  **Melcer** | 22 | 36 | NA | LT | NA | NA | YES | NO | YES | NO | NO | -right hydrocele.  -very small and hyperechoic left testicle | NO | 2640 | 9-10 | Conservative management. At 3 months  follow-up, the affected testicle demonstrated complete  atrophy and no color flow.  The contralateral testicle remained within the normal clinical size with good color flow on ultrasound. |
| **2006**  **Arena** | NA | 33 | NA | RT | NA  reduced | NO | YES | NA | YES | NO | NO | Hydrocele with a small hyperechoic testis | NO | NA | NA | Urgent orchiectomy and contralateral orchiopexy |
|  | NA | 34 | NA | LT | NA  enlarged | NO | NO | NO | NO | NO | NO | Enlarged and heterogeneous testis with surrounding hypoechoic rim and central hypoechoic area | NO | NA | NA | Urgent orchiectomy and contralateral orchiopexy |
|  | 28 | 34+5 | >40 | BLT | NA  enlarged | NO | YES | NO | YES | NO | NO | Bilateral hydrocele. Both testes were enlarged hyperechoic and heterogeneous | NO | 4100 | 9-10 | Emergency scrotal exploration revealed bilateral extravaginal torsion.  A bilateral derotation was performed  The patient was referred to a pediatric endocrinologist for medical therapy |
|  | NA | 33 | NA | LT | NA  enlarged | NO | YES | NO | YES | NO | NO | Heterogeneous, hyperechoic rim-like layer, hypoechoic central areas | NO | NA | NA | A biopsy through the scrotal route revealed a frankly necrotic testis |
| **2002**  **Herman** | 33 | 39 | NA | RT | NA | NO | NO | NO | YES | NO | NO | Hydrocele on one side of the scrotum and normal size testicle, the other hemiscrotum demonstrated enlarged testicle surrounded by two hypoechoic fluid concentric layers suggesting acute torsion with hemorrhage (‘double-ring hemorrhage’ sign) | NO | 3900 | 9-10 | Urgent orchiectomy and contralateral orchidopexy |
| **2001**  **Ricci** | 31 | 37 | 38 | RT | NA  enlarged | NO | YES | NO | YES | NO | NO | NO  Enlarged but homogeneous testicle with enlarged and hyperechoic epididymis | NO | 3370 | 8-9 | Urgent orchiectomy and contralateral orchiopexy |
| **2000**  **Youssef BA** | 30 | NA | >37 | LT | NA | NO | NO | NO | YES | NO | NO | -Enlarged heterogeneous left testicle with a central hypoechoic  area and peripheral hypoechoic rim. -Small right-sided  hydrocele. | NO | 3220 | 9-9 | Urgent orchiectomy and contralateral orchiopexy |
| **2000**  **Olguner** | 32 | 34 | 39 | RT | NA | NO | NA | NO | YES | NO | NO | NO | NO | 3700 | NA | Postnatal urgent scintigraphy revealed bilateral testicular torsion. Urgent right orchiectomy and contralateral detorsion with orchiopexy was performed |
| **1998**  **Devesa** | 27 | 39 | 40 | LT | 7X9  reduced | NO | YES | NO | NO | NO | NO | Rounded hypoechoic area within the left hemiscrotum with a peripheral  echogenic ring | NO | 3240 | 9-10 | Conservative management |
| **1995**  **Tripp** | 37 | 34 | 39 | BLT | NA | NO | YES | NO | NO | NO | NO | Bilateral hydroceles surrounded by a second fluid  layer bilaterally as well as bilateral inhomogenous testicles | NO | 3920 | 9-10 | Urgent surgery revealed bilateral extravaginal torsion of the spermatic cord |
|  |  |  |  |  |  |  |  |  |  |  |  |  |  |  |  |  |
| **1993**  **Gross** | 26 | 38 | NA | BLT | NA | NO | YES | NO | NO | NO | NO | NO  Heterogeneous right testis with hypoechoic center and bilateral  hydrocele | NO | NA | NA | Intraoperative findings of bilateral extravaginal testicular torsion |
| **1983**  **Hubbard** | 23 | 35 | 38 | RT | NA | NO | YES | NO | YES | NO | NO | NO  cystic swelling  containing a small solid sonolucent mass | NO | 4750 | NA | Delivery complicated by shoulder dystocia.  Fetal resuscitation was required with prompt response and good APGAR at 10 minutes  Left orchiopexy performed 8 months after birth |

***Abbreviations****: BLT = Bilateral, GA = gestational age, LT= Left, MRI= Magnetic Resonance Imaging, NA= Not Available, RT= Right*
